# Mechanical Force Modulates Alveolar Bone Marrow Mesenchymal Cells Characteristics for Bone Remodeling during Orthodontic Tooth Movement through Lactate Production

**DOI:** 10.3390/cells11233724

**Published:** 2022-11-22

**Authors:** Mingrui Zhai, Shuyue Cui, Lan Li, Chen Cheng, Zijie Zhang, Jiani Liu, Fulan Wei

**Affiliations:** Department of Orthodontics, School and Hospital of Stomatology, Cheeloo College of Medicine, Shandong University & Shandong Key Laboratory of Oral Tissue Regeneration & Shandong Engineering Laboratory for Dental Materials and Oral Tissue Regeneration, No.44-1 Wenhua Road West, Jinan 250012, China

**Keywords:** lactate, orthodontic tooth movement, epigenetic modifications, bone remodeling

## Abstract

Orthodontic tooth movement (OTM) relies on mechanical force-induced bone remodeling. As a metabolic intermediate of glycolysis, lactate has recently been discovered to participate in bone remodeling by serving as a signaling molecule. However, whether lactate could respond to mechanical stimulus during OTM, as well as whether lactate has an impact on the alveolar bone remodeling during orthodontics, remain to be further elucidated. In the current study, we observed physiologically elevated production of lactate along with increased osteogenic differentiation, proliferation, and migration of alveolar bone marrow mesenchymal cells (ABMMCs) under mechanical force. Inhibition of lactate, induced by cyclic mechanical stretch by GNE-140, remarkably suppressed the osteogenic differentiation, proliferation, and migration, yet enhanced apoptosis of ABMMCs. Mechanistically, these regulatory effects of lactate were mediated by histone lactylation. Taken together, our results suggest that force-induced lactate is involved in controlling bone remodeling-related cellular activities in ABMMCs and plays a vital role in the alveolar bone remodeling during OTM. Our findings indicate that lactate might be a critical modulator for alveolar bone remodeling during OTM, providing a novel therapeutic target for the purpose of more effectively controlling tooth movement and improving the stability of orthodontic results.

## 1. Introduction

Orthodontic tooth movement (OTM) relies on force-induced bone remodeling, in which bone is deposited on the tension side and absorbed on the compression side [1]. Bone mesenchymal stem cells (BMSCs) have been identified as a key component for bone remodeling during OTM [2]. Among these, alveolar bone marrow mesenchymal cells (ABMMCs) are a class of BMSCs that reside in the alveolar bone, with proliferation, migration, and osteogenic differentiation properties [3]. They have been proved to be mechanosensitive and able to sense and transduce mechanical signals into complex biological responses [4]. To our knowledge, various cellular activities and characteristics are associated with bone remodeling during OTM. The proliferation and survival of BMSCs, for instance, are responsible for bone growth and bone homeostasis during OTM [5]. In addition, the capability of migration enables BMSCs to move to the regions surrounding the moving teeth, which determines the velocity of tooth movement to a certain degree [6,7]. With the ability to differentiate into mature osteoblasts, BMSCs could directly participate in force-related bone remodeling [8]. Therefore, elucidating these bone remodeling-related cellular activities in ABMMCs under mechanical force, as well as their underlying mechanisms, is of critical importance for orthodontic therapies.

Bone remodeling triggered by mechanical force is a complex biological process that requires a large amount of energy for the synthesis of macromolecules (e.g., DNA, RNA, and proteins) related to the proliferation, differentiation, and function of cells [9]. Therefore, an extensive metabolic reconfiguration occurs during bone remodeling. To date, a variety of research has focused on the energy pathways and energy sources involved in bone remodeling [10]. Glucose has been recognized as the major source of energy for bone development and growth [11]. In addition, glycolysis has been proven to be the main pathway to meet the demanding energy for bone remodeling [12,13]. Additionally, stimulated glycolysis was observed under mechanical stimuli in the previous study, with increased glucose consumption and lactate generation [14,15]. In addition to its role as a well-known energy source, glycolysis also provided intermediates and substrates such as fructose 1,6-bisphosphate, pyruvate, and lactate for other metabolic pathways [16,17]. Moreover, it was recently reported that these intermediates could regulate various cellular activities and functions by serving as signaling molecules [18].

Lactate, as one of the above intermediates produced during glycolysis, has been discovered to exert multiple regulatory functions in both physiological and pathological conditions [19]. The production of lactate predominantly occurs in the cytoplasm, where it is derived from the conversion of pyruvate by lactate dehydrogenase A (LDHA). Through its transporters (monocarboxylate transporters) and receptors (G protein-coupled receptor 81), lactate is involved in the regulation of wound healing, endothelial cell migration, tumor development, and immune cell polarization [20]. Since lactate has been confirmed as the main product of glucose metabolism in osteoblasts, its role in bone formation and regeneration has been investigated recently [21,22]. For instance, Wu et al. reported a pro-osteogenesis effect of lactate at 5 mM in osteoblasts [21]. On the contrary, a higher concentration of lactate at 10 mM was revealed to inhibit the osteogenic differentiation of periodontal ligament stem cells (PDLSCs) via the MCT1-mTOR signaling pathway [22]. Generally, this is because the effects of lactate are largely dependent on the concentration as well as its subcellular localization [19]. It was documented that the physiological concentration of lactate was approximately 1.5–3 mM in blood and healthy tissues [23], but could rise to 10 mM in inflammatory sites and to almost 20–30 mM in cancer tissues [24]. However, it remains unknown whether lactate is altered by the mechanical force during OTM or the what the effects of lactate may be, at that concentration, on the bone remodeling.

In this study, we observed increased lactate generation along with enhanced osteogenic differentiation, proliferation, and migration in ABMMCs under mechanical force. Furthermore, we discovered that mechanical stretch-derived lactate was able to promote the osteogenic differentiation, proliferation, and migration, yet decrease the apoptosis, of ABMMCs via histone lactylation. Taken together, our study’s findings revealed the critical role of lactate as a mediator and regulator for alveolar bone remodeling during OTM, and started a series of studies in the field of the function of force-related metabolites.

## 2. Materials and Methods

### 2.1. Animals and Ethic Statement

All experiments on rats in the current study were carried out with the approval of the Committee on the Ethics of Animal Experiments of Shandong University (NO. 20210109). Healthy male 8-week-old Wistar rats (Charles River, Beijing, China) were used in this study. They were fed with a sterilized diet and kept at 25 °C at 60% humidity, with a 12-h artificial light/dark cycle.

The rat OTM model was established according to our previous research [25]. Briefly, the first left maxillary molars of rats were ligated to the upper incisors with 0.2 and nickel-titanium closed-coil springs (TOMY, Fukushima Prefecture, Japan). The stretch force was approximately 20 g. To fix the above appliance, light-curing resin (3 M, St. Paul, MN, USA) was applied at the upper incisors. The rats were sacrificed on day 3, 7, and 14 (*n* = 3 at each time). The whole maxillae were dissected and divided into the left and right parts for the following experiments. The right side was taken as the control.

### 2.2. Immunohistochemistry (IHC)

Isolated samples were decalcified and embedded for sectioning into 5 μm. Sections were deparaffinized and retrieved with antigen with 0.25% trypsin (Solarbio) for 30 min at 37 °C. Subsequently, 3% hydrogen peroxide was added for 15 min, and 5% normal goat serum (Solarbio) was added for block for 1 h at room temperature. The antibodies anti-LDHA (Abcam) and anti-lactyl-lysine (PTM Bio) were added to the sections overnight at 4 °C. The second antibody was added and then counterstained with hematoxylin (Solarbio). Notably, the distal side of the upper and middle third of mesiobuccal roots was considered the tension zone due to the tipping movement of the teeth. The quantification was performed using ImageJ software.

### 2.3. Isolation and Identification of ABMMCs

ABMMCs were isolated and cultured using previously described methods [26]. Mandibles without molars or soft tissue were obtained from 4-week-old Wistar rats, and then digested with 3 mg/mL collagenase I (Solarbio) and 4 mg/mL dispase II (Roche) at 37 °C for 1 h. Cells were then collected and cultured in primary medium containing α-minimal essential medium (α-MEM; BasalMedia), 15% fetal bovine serum (FBS; Lonsa Science Srl), and 10,000 U/mL penicillin-streptomycin (Biosharp). The ABMMCs used in this study were 3–5 generations.

Flow cytometry was used to identify the phenotype of ABMMCs. In brief, ABMMCs were resuspended with phosphate-buffered saline (PBS; BasalMedia). Then, CD44, CD31 (BD; Biosciences), CD90, and CD45 (Elabscience) antibodies were applied to detect the phenotype of ABMMCs as directed by the manufacturer.

### 2.4. Osteogenic Differentiation and Adipogenic Differentiation of ABMMCs

Briefly, ABMMCs were cultured with osteogenic-induction medium (OM) containing 50 μg/mL vitamin C, 10 M β-glycerophosphate, and 10 nM dexamethasone (Sigma-Aldrich, Shanghai, China) [27]. After osteogenic induction for 7 days, the ALP staining test was conducted to detect calcium deposition using the ALP staining kit (Beyotime Biotechnology), according to the manufacturer’s protocol. After osteogenic induction for 21 days, cells were stained with 1% Alizarine Red (pH = 4.2; Sigma-Aldrich, Shanghai, China) to detect the mineralized nodules.

ABMMCs were cultured with adipogenic-induction medium (AM) containing 0.5 M 3-isobutyl-1-methylxanthine, 10 μg/mL insulin, 0.2 mM indomethacin, and 1 μM dexamethasone [28]. After adipogenic induction for 21 days, cells were fixed and stained with Oil Red O (Solarbio) to detect the lipid droplets.

### 2.5. Application of Cyclic Mechanical Stretch

Flexcell amino silicone rubber plates coated with collagen type I (Collagen I, rat tail, Corning, NY, USA) were used to culture ABMMCs. Cells were subjected to cyclic mechanical stretch (10% elongation, 0.5 Hz; sinusoidal waveforms) for 6, 12, and 24 h by the Flexcell-FX-6000-Tension System. The control group was cultured under the same conditions without stretching.

### 2.6. Lactate Assay

The level of lactate was detected using the Lactate Content Assay Kit (Solarbio). As directed by the manufacturer, both the supernatants and lysates of cultured cells were harvested to detect the extracellular and intracellular lactate content, respectively. Then, the homogenates of the alveolar bone on the tension side of the first molar were also harvested according to the manufacturer’s protocol in order to measure the lactate concentration in vivo. Finally, the absorbance was detected on the microplate reader.

### 2.7. Cell Cycle Assessment

Cells were cultured in a medium without serum for 24 h prior to treatment to achieve a quiescent state. The culture medium was then changed to a growth medium containing 10% FBS for indicated treatments. The cell cycle distribution was tested using the Quantitative DNA Content Assay Kit (cell cycle) (Solarbio). Briefly, the cells were collected and fixed with 70% ethanol overnight at 4 °C. Following this, 100 μL RNase A solution and 400 μL PI solution were added and incubated in the dark for 30 min. Then the fluorescence was detected at 488 nm by the flow cytometer (BD FACSCalibur, Milan, Italy).

### 2.8. Hoechst 33,258 Staining

Following fixing, the samples were stained with the Hoechst Staining Kit (Beyotime Biotechnology). Apoptosis was observed using a fluorescent inverted phase-contrast microscope (TH4-200; Olympus, Tokyo, Japan). The apoptosis of cells was identified by morphological alterations, including chromatic agglutination and nuclear fragmentation [29].

### 2.9. Annexin V-FITC/PI Assay

Briefly, the collected cells were washed, suspended in 195 μL 1 × binding buffer, and incubated with Annexin V-FITC and/or PI (Dojindo) for 15 min at room temperature. The samples were then analyzed using the flow cytometer. The apoptotic rate was analyzed as the proportion of Annexin V-positive cells.

### 2.10. RNA Extraction and Real-Time Polymerase Chain Reaction

Total RNA was extracted using RNAiso TM Plus (Takara, Shiga, Japan). Reverse transcription to complementary DNA was performed using the Prime Script RT Reagent Kit (Takara) according to the manufacturer’s protocol. Then, the quantification of the relative transcription level, using SYBR^®^ Premix Ex Taq™ (Takara), was normalized to the transcription level of β-actin. The primer sequences are shown in Table 1.

### 2.11. Western Blot (WB)

Total protein was extracted and lysed in RIPA reagent (Solarbio) with 1% PMSF (Solarbio). Specifically, the nuclear protein was extracted using the Nuclear Protein Extraction Kit (Solarbio). Then, the lysates were separated on 10% or 15% sodium dodecyl sulfate-polyacrylamide gels (SDS-PAGE) following transfer to a polyvinylidene fluoride membrane (PVDF; Millipore). After blocking with 5% skimmed milk, these membranes were incubated with the following primary antibodies: anti-RUNX2 (Cell Signaling Technology), anti-ALP (Proteintech Group, Inc.), anti-LDHA (Abcam), anti-β actin (Abcam), anti-Histone 3 (Abcam), and anti-lactyl-lysine (PTM Bio) overnight at 4 °C. Following washing with Tris-buffered saline containing 0.05% Tween 20, the second antibody was added and incubated for 1 h. The visualization was performed with the ECL chromogenic substrate (Millipore).

### 2.12. GNE-140 Application

For GNE-140 application, we dissolved GNE-140 (MedChemExpress) in DMSO (Solarbio) to form a 4-mM stock solution. Before the cyclic mechanical force application, cells were incubated with GNE-140 (10 μM) or DMSO (10 μM) for 24 h.

### 2.13. Pyruvate Detection

The level of pyruvate was detected using the Pyruvate assay kit (Nanjing Jiancheng Bioengineering Institute, Nanjing, China). Briefly, we isolated the sample according to the manufacture’s instruction, and the OD value was detected at 450 nm.

### 2.14. Micro-CT Scanning

The micro-CT (Quantum GX2) was used to scan the samples. The scan setting was set with a standard acquisition protocol (90 kV, 88 μA, and 72 μM voxel size). CTAn (Skyscan) software was used to reconstruct images from the sagittal view.

### 2.15. Statistical Analysis

All data came from at least three separate experiments. Data were presented as mean ± standard deviation (SD). Statistical analyses were performed with GraphPad Prism 8. All data in our study were normally distributed. Comparisons between the two groups were performed using Student’s t-tests. For multiple groups, data were analyzed by analysis of variance (ANOVA), and the Bonferroni test was used for pairwise comparisons. *p* < 0.05 was identified as statistically significant.

## 3. Results

### 3.1. Isolated ABMMCs Maintained Stem Cell Properties and Cyclic Stretch Promoted Their Osteogenic Differentiation

**Figure 1:**
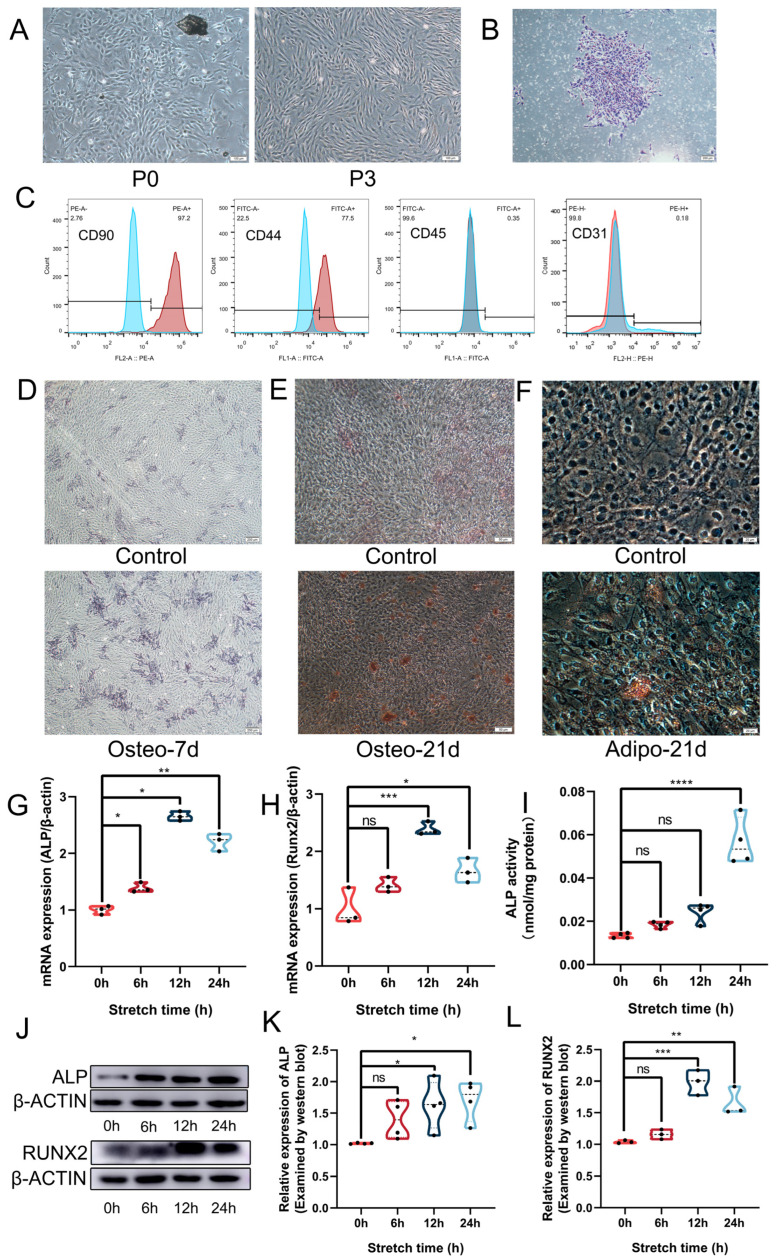
Isolated alveolar bone marrow mesenchymal cells (ABMMCs) maintained stem cell properties, and cyclic stretch promoted their osteogenic differentiation. (**A**) Morphology of ABMMCs at passage 0 and passage 3; scale bar = 100 μm. (**B**) A single cellular cluster of ABMMCs via crystal violet staining; scale bar = 200 μm. (**C**) The surface antigens of ABMMCs analyzed by flow cytometry. (**D**) The ALP staining of ABMMCs after osteogenic induction for 7 days; scale bar = 200 μm. (**E**) Alizarin Red S staining of ABMMCs after osteogenic induction for 21 days; scale bar = 200 μm. (**F**) Oil Red O staining of ABMMCs after adipogenic induction for 21 days; scale bar = 50 μm. (**G**,**H**) The expression of ALP and RUNX2 at the mRNA level in ABMMCs after being stretched for 0, 6, 12, and 24 h. (**I**) The ALP activity in ABMMCs after being stretched for 0, 6, 12, and 24 h. (**J**–**L**) The expression of ALP and RUNX2 at the protein level in ABMMCs after being stretched for 0, 6, 12, and 24 h. * *p* < 0.05. ** *p* < 0.01. *** *p* < 0.001. **** *p* < 0.0001. ns = no significance.

Rat ABMMCs were in spindle or triangle shapes (Figure 1A), and they demonstrated the ability to form cell clusters in the clone formation test (Figure 1B). As demonstrated in Figure 1C, the ABMMCs expressed MSC-specific positive markers (CD 90 and CD 44) and failed to express MSC negative markers (CD 31 and CD 45). Moreover, enhanced calcium deposition and mineralized nodules after osteogenic induction was observed in ALP staining and Alizarin Red S staining, respectively, demonstrating the osteogenic differentiation potential of ABMMCs (Figure 1D,E). Oil Red O staining revealed that ABMMCs have the potential for adipogenic differentiation (Figure 1F). We then assessed the effect of mechanical stimuli on the osteogenic differentiation of ABMMCs. Since there might be post-transcriptional regulations in genes, we assessed both the gene transcriptional and translational level under cyclic mechanical stretch. The expression of osteogenesis-associated genes (ALP and RUNX2) began to increase at 6 h and reached the peak at 12 h (Figure 1G,H). Meanwhile, the expression of ALP and RUNX2 at the protein level was also significantly elevated by mechanical force (Figure 1J–L). Moreover, the cellular ALP enzyme activity of ABMMCs was substantially increased after cyclic tension for 24 h (Figure 1I). The above findings confirmed ABMMCs’ multiple differentiation potential and clone formation ability, indicating that ABMMCs were self-renewable and pluripotent cells. We also confirmed promoted osteogenic differentiation of ABMMCs under cyclic mechanical stretch.

### 3.2. Mechanical Force Increased the Expression of LDHA and Lactate Production

**Figure 2:**
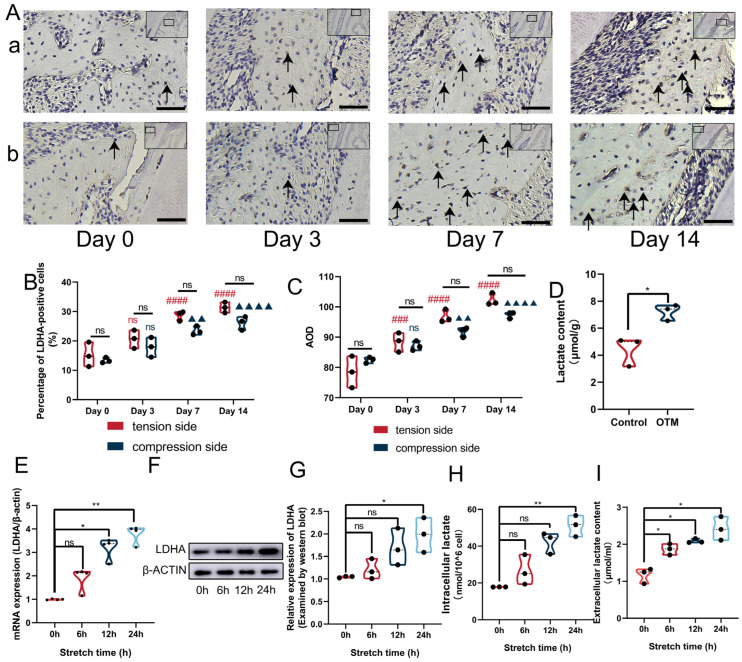
Mechanical force increased the expression of lactate dehydrogenase A (LDHA) and lactate production. (**A**–**C**) The expression of LDHA at the tension side (**a**) and the compression side (**b**) during OTM, as determined by immunohistochemistry (IHC). The black arrows indicated positive cells; scale bar = 50 μm. The expression of LDHA was determined by both the percentage of LDHA-positive cells (B) and the average optical density (AOD) (**C**) (### and #### represented *p* < 0.01 and *p* < 0.0001 in comparison with Day 0 on the tension side, respectively; ▲▲ and ▲▲▲▲ represented *p* < 0.01 and *p* < 0.0001 in comparison with Day 0 on the compression side, respectively; ns represented no significance between the tension side and the compression side). (**D**) The lactate content in the alveolar bone after being subjected to orthodontic force for 7 days. (**E**) The expression of LDHA at the mRNA level after being stretched for 0, 6, 12, and 24 h. (**F**,**G**) The expression of LDHA at the protein level after being stretched for 0, 6, 12, and 24 h. (**H**) Intracellular lactate concentration after being stretched for 0, 6, 12, and 24 h. (**I**) Extracellular lactate concentration after being stretched for 0, 6, 12, and 24 h. * *p* < 0.05. ** *p* < 0.01. ns = no significance.

In order to assess the expression of LDHA and lactate production during OTM in vivo, we established the OTM model in rats and observed mesial movement of the first left maxillary molars (Supplementary Figures S1 and S2). The results of IHC revealed increased expression of LDHA on both tension and compression sides in the alveolar bone after applying orthodontic force for 7 days, with the increased percentage of LDHA positive cells as well as increased average optical density (AOD) (Figure 2A–C). The expression of LDHA was slightly higher on the tension side as compared to the compression side, while no significant difference was observed between the two sides (Figure 2A–C). Consistently, the lactate concentration in the alveolar bone surrounding the moved molars was also upregulated after 7 days of OTM (Figure 2D). However, limited by the amount of alveolar bone available around the moved molars, the detection of LDHA expression by WB was not achieved in the current study. In terms of findings in vitro, we observed remarkably elevated expression of LDHA at both the mRNA and the protein level in ABMMCs after 12 h of cyclic tension (Figure 2E–G). As shown in Figure 2H,I, intracellular lactate was increased by around 2.5-fold, and extracellular lactate reached 2–3 mM after cyclic mechanical stretch for 24 h. In summary, mechanical force could promote the expression of LDHA and lactate production both in vivo and in vitro.

### 3.3. GNE-140 Inhibited the Osteogenic Differentiation of ABMMCs under Cyclic Mechanical Stretch

**Figure 3:**
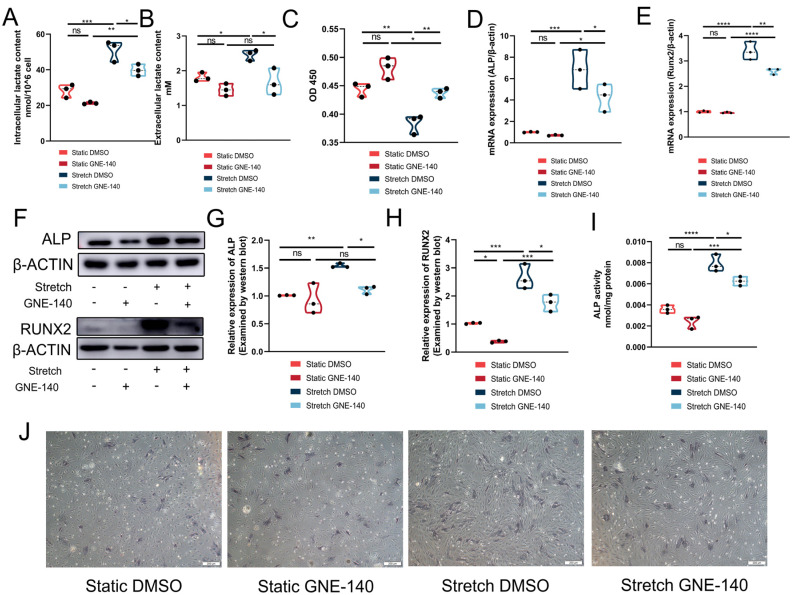
GNE-140 inhibited the osteogenic differentiation of ABMMCs under cyclic mechanical stretch. (**A**) Intracellular lactate concentration in GNE-140/DMSO-treated groups with or without cyclic mechanical stretch. (**B**) Extracellular lactate concentration in GNE-140/DMSO-treated groups with or without cyclic mechanical stretch. (**C**) Pyruvate level in GNE-140/DMSO-treated groups with or without cyclic mechanical stretch. (**D**,**E**) The expression of ALP and RUNX2 at the mRNA level in GNE-140/DMSO-treated groups with or without cyclic mechanical stretch. (**F**–**H**) The expression of ALP and RUNX2 at the protein level in GNE-140/DMSO-treated groups with or without cyclic mechanical stretch. (**I**) The ALP activity in GNE-140/DMSO-treated groups with or without cyclic mechanical stretch. (**J**) The ALP staining in GNE-140/DMSO-treated groups with or without cyclic mechanical stretch; scale bar = 200 μm. * *p* < 0.05. ** *p* < 0.01. *** *p* < 0.001. **** *p* < 0.0001. ns = no significance.

In order to investigate the effects of mechanical force-induced lactate on the osteogenic differentiation of ABMMCs, we applied GNE-140, a specific LDHA inhibitor, to inhibit the production of lactate during cyclic mechanical tension. Exposure to GNE-140 for 24 h during the cyclic mechanical tension resulted in a significant reduction in intracellular and extracellular lactate in ABMMCs, though no statistically significant inhibitory effects were found under static condition (Figure 3A,B). Moreover, the above results were reconfirmed by detecting the pyruvate level which was the upstream molecule of lactate (Figure 3C). The elevated expression of osteogenic markers (ALP and RUNX2) due to physiological mechanical force was remarkably attenuated by GNE-140 at both the gene transcription level (Figure 3D,E) and the protein translation level (Figure 3F–H). Congruently, enhanced intracellular ALP activity and mineralized nodules in ABMMCs due to cyclic mechanical stretch were attenuated by GNE-140 (Figure 3I,J). These results demonstrated that the enhanced osteogenic differentiation of ABMMCs under mechanical force could be suppressed by inhibiting lactate production. With these results taken together, mechanical force-induced lactate could promote the osteogenic differentiation of ABMMCs.

### 3.4. GNE-140 Suppressed the Proliferation and Migration Yet Promoted Apoptosis of ABMMCs under Cyclic Mechanical Stretch

**Figure 4:**
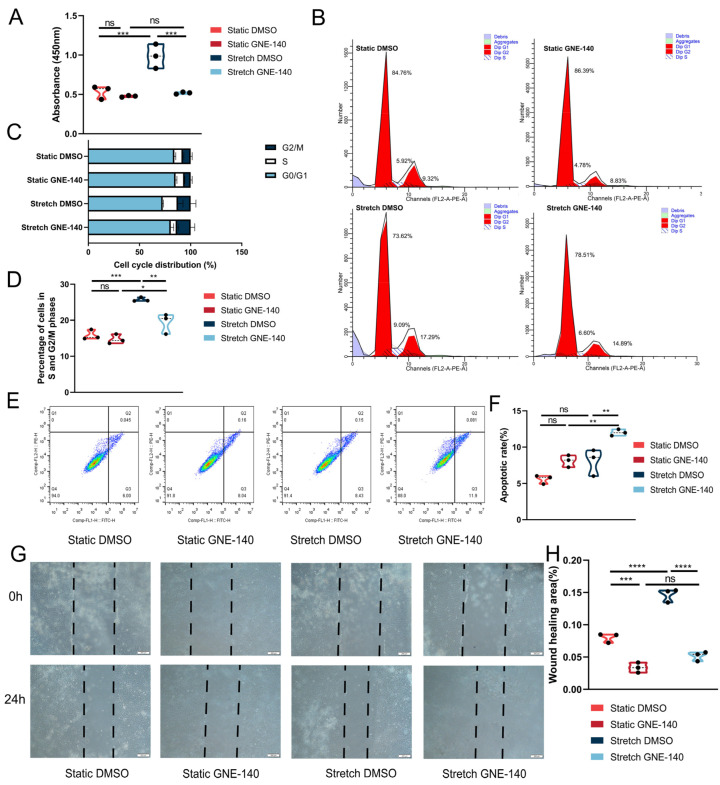
GNE-140 suppressed the proliferation and migration, yet promoted apoptosis, of ABMMCs under cyclic mechanical stretch. (**A**) The proliferation of ABMMCs determined by the CCK-8 test in GNE-140/DMSO-treated groups with or without cyclic mechanical stretch. (**B**–**D**) The cell cycle distribution (**B**), the percentage of cell cycle distribution (**C**), and the percentage of S and G2/M phases (**D**) in ABMMCs in GNE-140/DMSO-treated groups with or without cyclic mechanical stretch. (**E**,**F**) The Annexin V-FITC/PI double staining in ABMMCs in GNE-140/DMSO-treated groups with or without cyclic mechanical stretch. (**G**,**H**) Representative images (**G**) and the wound healing area (**H**) of the scratch assay in ABMMCs in GNE-140/DMSO-treated groups with or without cyclic mechanical stretch; scale bar = 200μm. * *p* < 0.05. ** *p* < 0.01. *** *p* < 0.001. **** *p* < 0.0001. ns = no significance.

We also evaluated the effects of lactate induced by mechanical force on other bone remodeling-related cellular activities, including the proliferation, apoptosis, and migration of ABMMCs. The proliferation of ABMMCs was promoted after 24 h of cyclic stretch treatment, and this increased proliferation rate was markedly attenuated by GNE-140 (Figure 4A). Meanwhile, physiological cyclic stretch could induce the cell cycle progression, as evidenced by the increased proportion of cells in the S and G2/M phases, while this trend was significantly hampered by GNE-140 (Figure 4B–D). In terms of apoptosis, no significant alteration was observed in ABMMCs under mechanical force, while GNE-140 significantly increased the apoptosis of ABMMCs, as detected by Annexin V-FITC/PI double staining (Figure 4E,F). In addition, the results of Hoechst staining (Supplementary Figure S3) also supported the notion that GNE-140 promoted the apoptosis of ABMMCs under mechanical force. Moreover, the migration of ABMMCs was remarkably induced by physiological cyclic stretch, with more cells migrating into the scratch area in the stretch group (Figure 4G,H). As demonstrated in Figure 4G,H, GNE-140 could abolish this pro-migration effect of mechanical force in ABMMCs. These results demonstrated that physiological lactate induced by mechanical force promoted the proliferation and migration, yet inhibited the apoptosis, of ABMMCs. In summary, these findings indicated that lactate had a vital function during bone remodeling by regulating cellular characteristics of ABMMCs.

### 3.5. Histone Lactylation Mediated the Regulatory Effects of Lactate on ABMMCs

**Figure 5:**
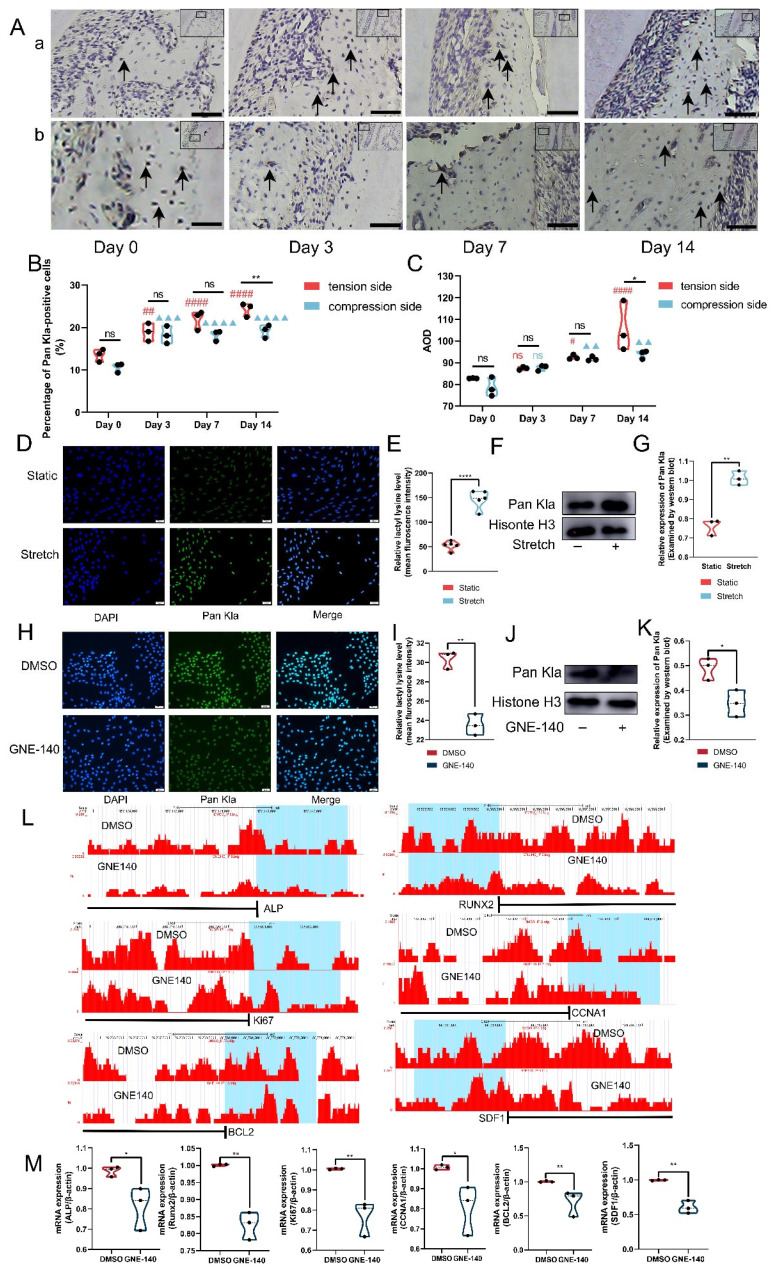
Histone lactylation mediated the regulatory effects of lactate on ABMMCs. (**A**,**B**) The level of histone lactylation determined by IHC on the tension side (**a**) and the compression side (**b**) during OTM. Arrows indicate positive cells; scale bar = 50μm. The level of histone lactylation was determined by the percentage of Pan-Kla positive cells (**B**) and the AOD (**C**). (#, ## and #### represent *p* < 0.05, *p* < 0.01 and *p* < 0.0001 in comparison with Day 0 on the tension side, respectively; ▲▲, ▲▲▲ and ▲▲▲▲ represent *p* < 0.01, *p* < 0.001 and *p* < 0.0001 in comparison with Day 0 on the compression side, respectively; * and ** represents *p* < 0.05 and *p* < 0.01 when comparing the tension side and the compression side) (**D**,**E**) The level of lactylated histone determined by IF with or without cyclic stretch. (**F**,**G**) The level of lactylated histone determined by WB with or without cyclic stretch. (**H**,**I**) The level of lactylated histone determined by IF in GNE-140/DMSO-treated groups. (**J**,**K**) The level of lactylated histone determined by WB in GNE-140/DMSO-treated groups. (**L**) A genomic snapshot of histone lactylation Chromatin immunoprecipitation sequencing (CHIP-seq) signal at related genes’ loci; scale bar = 2 kb. (**M**) The expression of represented genes at the mRNA level in DMSO/GEN-140 treated-groups. * *p* < 0.05. ** *p* < 0.01. **** *p* < 0.0001.

A recent study discovered a novel histone modification, referred to as histone lactylation, which participated in the reparative transition of macrophages [30]. Since lactate was the substrate for histone lactylation, we investigated whether the aforementioned effects of lactate were mediated by histone lactylation. The level of lactylated histone was up-regulated at both the tension and compression sides in the alveolar bone during OTM. In addition, the expression level of lactylated histone was obviously higher in the tension side compared to the compression side (Figure 5A–C). Meanwhile, the results of immunofluorescence (IF) and WB also confirmed the elevated level of lactylated histone in ABMMCs after cyclic stretch for 24 h (Figure 5D–G). These results implied a similar alteration pattern between lactylated histone and lactate generation under mechanical force. Next, we analyzed the relationship between histone lactylation and lactate production. Compared to the control group (DMSO), GNE-140 significantly decreased the level of lactylated histone (Figure 5H–K). Furthermore, the Chromatin immunoprecipitation sequencing (CHIP-sequence) results revealed suppressed enrichment of lactylated histone in the region that was 2 kilobases (kb) upstream of the transcription start site (TSS) of represented genes in the GNE-140-treated group (Figure 5L). The results of RT-qPCR also further verified decreased expression of these genes by GNE-140 in ABMMCs (Figure 5M). These findings indicated that the regulatory effects of mechanical force-derived lactate on bone remodeling were mediated by histone lactylation. Therefore, histone lactylation was verified as a critical mediator for lactate to achieve its regulating functions in alveolar bone remodeling during OTM.

## 4. Discussion

OTM relies on mechanical force-stimulated bone remodeling. Various cellular processes are involved in force-related bone remodeling, including proliferation, apoptosis, migration, and differentiation. Lactate has recently received significant attention as a signaling molecule for regulating cellular functions and activities [20]. However, whether lactate is affected by mechanical stimulus, as well as its effects on bone remodeling during OTM, require further investigation. In this study, we isolated ABMMCs from rat mandibles and observed enhanced production of lactate, as well as promoted proliferation, migration, and osteogenic differentiation of ABMMCs under mechanical force. Furthermore, inhibiting the lactate significantly suppressed the above cellular activities induced by mechanical force. Mechanistically, the regulatory effects of lactate were mediated by histone lactylation. Our results implied that lactate was a mediator and a regulator for bone remodeling during OTM, and confirmed its role in bone remodeling-related cellular characteristics. The present study indicated a novel potential strategy for regulating metabolic intermediates to control the alveolar bone remodeling during OTM.

ABMMCs were BMSCs that resided in the alveolar bone, and they were sensitive to mechanical stimuli. Until now, various cellular activities of BMSCs related to bone remodeling have been revealed to be modulated by mechanical stimuli [5]. The osteogenic differentiation of BMSCs, for instance, was facilitated by tensile force for bone generation and maintenance during OTM [3,8]. In addition, a previous study demonstrated elevated proliferation of BMSCs by mechanical force in a time- and strength-dependent manner for maintaining bone homeostasis [31]. The migration of BMSCs, associated with tooth movement velocity, was also induced by cyclic stretching via the FAK-ERK1/2 signaling pathway, according to Zhang et al. [32]. In the present study, we applied cyclic tensile force to ABMMCs at 10% elongation and 0.5 hz for 24 h as the physiological force, in order to mimic the orthodontic force according to our previous investigation [27]. Additionally, we observed elevated osteogenic differentiation, proliferation, and migration of ABMMCs under physiological mechanical stretch, confirming the critical role of ABMMCs in bone remodeling during OTM. Our findings have extended the understanding of bone remodeling-related cellular activities during cyclic mechanical stretch in ABMMCs.

Energetic pathways and metabolites were able to respond to mechanical signals [33,34]. Stiffness in the extracellular matrix, for instance, was disclosed to stimulate glycolysis through the cytoskeleton [15]. In addition, lactate was considered a potential mediator for cellular mechanical signals in PDLSCs, as it was closely linked to integrin-linked kinase (ILK), which was a key kinase in mechanical signal transduction [34]. Our results proved the presence of elevated lactate, both in vitro and in vivo, under mechanical force. Specifically, the concentration of extracellular lactate reached 2–3 mM after cyclic tension for 24 h. Compared to the concentration of lactate in inflammatory sites (10 mM) and tumor tissues (30 mM), lactate induced by mechanical force in our study was considered to be on a physiological level [35,36]. In fact, lactate has been revealed to accumulate in various conditions and tissues [37,38,39]. Additionally, lactate could modulate the metabolic pathways, immune responses, and cell-to-cell communication through various targets, including post-translational modifications, G-protein coupled receptors, and transcriptional factor activation such as NF-κB and HIF-1α [37]. Therefore, lactate elevated under mechanical force could only be regarded as a potential mediator and modulator for OTM instead of a biomarker, since it was not specific to orthodontic processes. With these results taken together, lactate was elevated during cyclic mechanical stretch, indicating that lactate might be a critical modulator for bone remodeling during OTM.

After confirming the physiologically upregulated lactate under mechanical force, we explored the effects of lactate at this level on bone remodeling-related cellular characteristics of ABMMCs. To date, a variety of studies have been conducted to investigate the effects of lactate on cellular activities in different tissues, although the standpoints were substantially controversial [20]. In terms of osteogenic differentiation, 5 mM lactate could promote the osteoblast differentiation by stabilizing HIF-1α, yet a higher level of lactate exerted inhibitory effects on osteogenesis in PDLSCs [21,22]. Similarly, the effect of lactate on migration also depended on its concentration. Beckert et al. [40] found a migration-promoting function of lactate at physiological concentrations in endothelial cells. However, Goetze et al. [41] confirmed a negative effect of lactate at 20 mM on monocyte migration. Moreover, it was claimed that lactate could promote the proliferation of tumor cells, whereas it suppressed the viability and increased the apoptosis of immune effector cells [42,43]. Therefore, the functions of lactate not only depended on its concentrations, but also on cell types. In this study, we applied GNE-140 in order to inhibit lactate production under mechanical force. We also observed that GNE-140 only produced an inhibitory effect on lactate production under cyclic tension. This might be interpreted as stating that the activity of LDHA under static conditions was low, providing limited active sites with which GNE-140 could bind and form hydrogen bonds [44]. Thus, neither lactate levels nor pyruvate levels were significantly changed without stretch stimulation. In contrast, under mechanical stretch, GNE-140 suppressed the lactate production and inhibited the osteogenic differentiation, proliferation, and migration, as well as, on the other hand, the decrease in the apoptosis of ABMMCs. Collectively, our findings indicated that mechanical force-derived lactate participated in the regulation of various bone remodeling-related cellular activities.

With rapid clearance in vivo, GNE-140 was not able to inhibit lactate production after 1 h of application [44], inhibiting its application in vivo. Similar characteristics regarding the rapid clearance in vivo were also found in lactate [45]. Therefore, current studies investigating the effects of GNE-140 were restricted to the cellular level [44,46,47]. To some extent, our in vitro findings that GNE-140 suppressed the proliferation, migration, and osteogenic differentiation of ABMMCs under mechanical force could provide cellular evidence and theoretical basis for future in vivo experiments. Future investigations applying GNE-140 in combination with sustained drug release systems, or using more stable lactate inhibitors with a favorable in vivo tolerability, biodistribution, and bioavailability profile, would be required to confirm our findings in vivo.

**Scheme 1:**
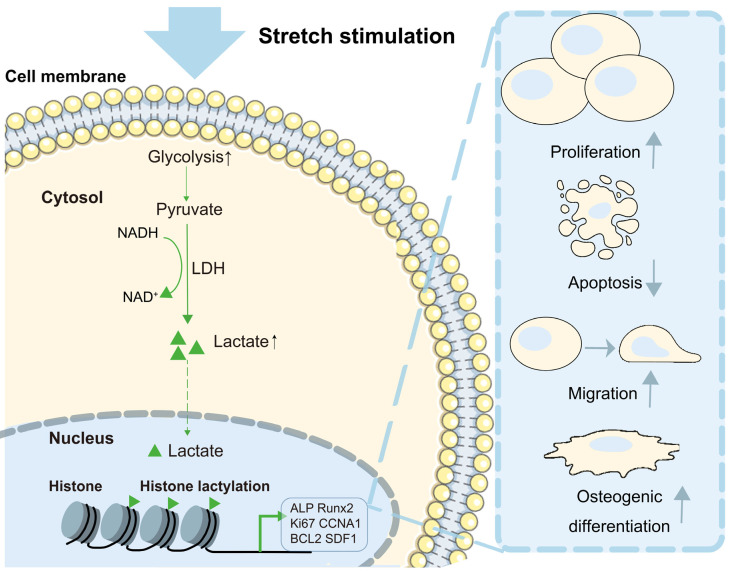
Stretch-stimulated lactate promoted the expression of ALP, Runx2, Ki67, CCNA1, BCL2, and SDF1 in ABMMCs through histone lactylation. Under stretch stimulation, the production of lactate could be elevated in ABMMCs. The stretch-induced lactate further promoted the proliferation, migration, and osteogenic differentiation of ABMMCs through histone lactylation of related genes. ABMMCs: alveolar bone marrow mesenchymal cells; NADH: nicotinamide adenine dinucleotide; LDH: lactate dehydrogenase.

It has been widely documented that metabolic substances could regulate transcriptional responses at various levels, including epigenetic modulations [48,49]. According to Zhang et al., lactate could elevate the level of histone lactylation enriched at the promotor of arginase-1 (Arg1) and further increase the expression of Arg1 at the transcriptional level [30]. In this study, we confirmed a coordinate pattern of lactate production and histone lactylation during force-induced bone remodeling. Moreover, we further revealed the mediatory role of histone lactylation in the aforementioned regulatory effects of lactate. Interestingly, various lactylated proteins have been identified in a recent report by Gaffney et al. [50] However, whether or not bone remodeling-related proteins or enzymes were lactylated by mechanical force-derived lactate remains to be further investigated. Collectively, our findings revealed that the regulatory effects of lactate in alveolar bone remodeling could be mediated by histone lactylation (Scheme 1).

## 5. Conclusions

In conclusion, our study confirmed the presence of physiologically elevated lactate in response to mechanical force during OTM. Moreover, the role of lactate in the regulation of bone remodeling-related cellular activities and characteristics of ABMMCs was revealed. Our research identified lactate as a potential modulator for alveolar bone remodeling during OTM, providing a novel therapeutic target for improving the remodeling of alveolar bone during orthodontics.

## Figures and Tables

**Table 1:** The primer sequences used in this study.

Primer Name	Forward Primers (5′–3′)	Reverse Primers (3′–5′)
ALP	ACAACACCAACGCTCAGGTC	GTGACCTCGTTCCCCTGAGT
RUNX2	ACGAATGCACTATCCAGCCA	GCAGGTACGTGTGGTAGTGA
LDHA	CCTCAGCGTCCCATGTATCC	TCTGCACTCTTCTTCAGGCG
Ki67	TATCCAGCTGCCTGTAGTGT	AGGAGCGGTCAATGATGGT
CCNA1	GCTTGTCAGATGCTCACCCG	GCCCTCCTGTACTGCTCATTT
BCL2	TGGCCTTCTTTGAGTTCGGT	GTTCCACAAAGGCATCCCAGC
SDF-1	TACAGATGCCCCTGCCGATT	CTGAAGGGCACAGTTTGGAGT
β-ACTIN	CTCTGTGTGGATTGGTGGCT	CGCAGCTCAGTAACAGTCCG

## Data Availability

The data presented in this study are available upon request from the corresponding author.

## Supplementary Materials

The following supporting information can be downloaded at: https://www.mdpi.com/article/10.3390/cells11233724/s1, Figure S1: Micro-CT of control group and orthodontic tooth movement groups at day 3, 7, and 14; Figure S2: Hematoxylin and eosin staining of control group and orthodontic tooth movement groups at day 3, 7, and 14; Figure S3: Hoechst staining of ABMMCs in GNE-140/DMSO-treated groups with or without cyclic mechanical stretch. Upper panel: scale bar = 200μm; lower panel: scale bar = 50μm.
