# Supplementary material for: Mechanical Force Modulates Alveolar Bone Marrow Mesenchymal Cells Characteristics for Bone Remodeling during Orthodontic Tooth Movement through Lactate Production
Source: Cells. 2022 Nov 22;11(23):3724. doi: 10.3390/cells11233724 (PMC9738738; doi:10.3390/cells11233724)
Supplement: Supplementary file 1 [file cells-11-03724-s001.zip › cells-1937638-supplementary.pdf]

Supplement Figures

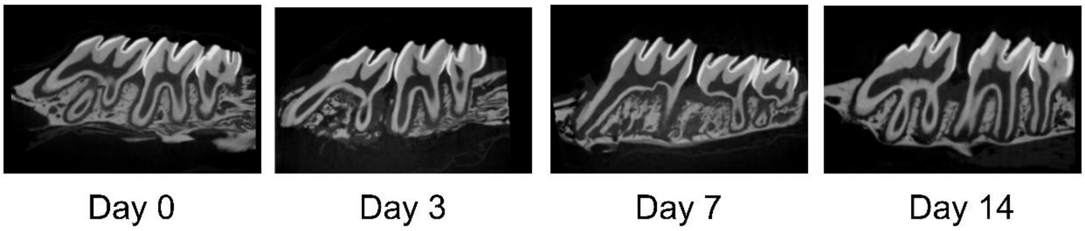

Figure S1. Micro-CT of control group and orthodontic tooth movement groups at day 3, 7, and 14.

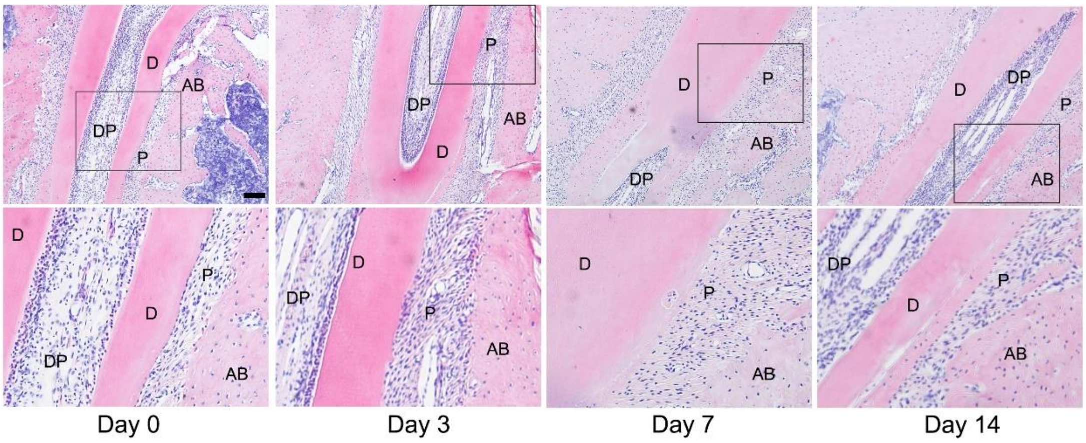

Figure S2. Haematoxylin and eosin staining of control group and orthodontic tooth movement groups at day 3, 7, and 14. D, dentin; DP, dental pulp; P, periodontal ligament; AB, alveolar bone.

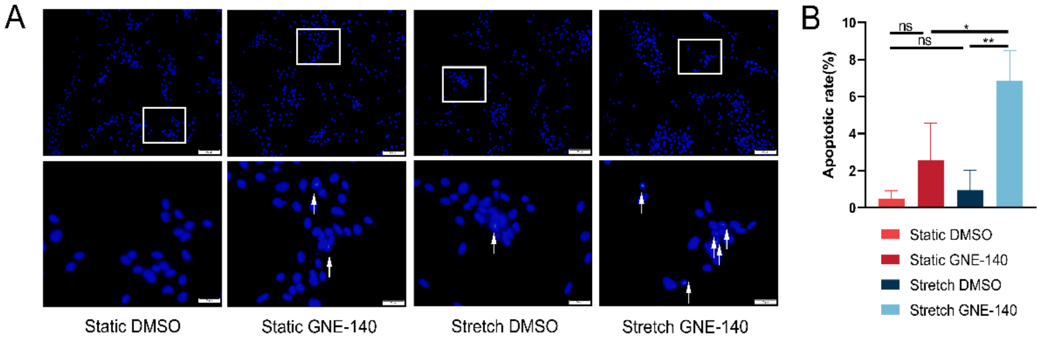

Figure S3. Hoechst staining of ABMMCs in GNE-140/DMSO treated groups with or without cyclic mechanical stretch. Upper panel: scale bar=200μm; Lower panel: scale bar=50μm.
